# Isothermal and Non-Isothermal Crystallization Kinetics of Poly(ethylene chlorotrifluoroethylene)

**DOI:** 10.3390/polym14132630

**Published:** 2022-06-28

**Authors:** Xiaodong Yang, Bin Yu, Hui Sun, Nan Wang, Peng Liu, Jiangli Feng, Xiaogang Cui

**Affiliations:** Zhejiang Provincial Key Laboratory of Fiber Materials and Manufacturing Technology, College of Textiles Science and Engineering, Zhejiang Sci-Tech University, Hangzhou 310018, China; zstuxiaodongyang@163.com (X.Y.); 18846049747@163.com (N.W.); lp18848862271@163.com (P.L.); 18758094708@163.com (J.F.); c503950252@163.com (X.C.)

**Keywords:** poly(ethylene chlorotrifluoroethylene), crystallization kinetics, crystallization activation energy, morphology, structure

## Abstract

The isothermal (IT) and non-isothermal (NIT) crystallization kinetics, morphology, and structure of poly(ethylene chlorotrifluoroethylene) (ECTFE) were investigated via differential scanning calorimetry (DSC), polarized optical microscopy (POM), and wide-angle X-ray diffraction (XRD). The Avrami equation could well describe the overall IT crystallization process of ECTFE, and, furthermore, the overall crystallization rate decreased at higher crystallization temperatures (*T_c_*). The equilibrium melting point for ECTFE was found to be 238.66 °C. The activation energies for IT and NIT crystallization were determined as −137.68 and −120.54 kJ/mol, respectively. The Jeziorny model fitted well with the initial stages of NIT melt crystallization, while deviations from linearity in the later stages of the process were due to the collisions of spherulites. Spherulites of ECTFE organized in a hexagonal crystal system were found. The relative crystalline degree of ECTFE under NIT conditions was about 54.55%, and this decreased with the increase in cooling rate. Moreover, the Ozawa and Mo models were suitable for modeling the overall NIT crystallization process of ECTFE.

## 1. Introduction

Poly(ethylene chlorotrifluoroethylene) (ECTFE) [1,2,3] is a functional copolymer composed of ethylene and chlorotrifluoroethylene. This fluoropolymer with the repeated unit of (CH_2_-CH_2_-CFCl-CF_2_)*_n_* is already on market under the brand name HALAR^®^. The polymer structure comprises of perfectly alternating monomers, which are beneficial for its high crystallinity [4]. Moreover, a high crystalline degree can be ascribed to the restricted conformational mobility of a polymer due to the polarity of C–F bonds and the larger Van der Waals radii of fluorine and chlorine compared to hydrogen atoms [5,6].

The semi-crystalline nature of ECTFE endows the polymer with outstanding mechanical strength, thermal stability (>150 °C), chemical resistance, and durability in strongly acidic, alkaline, and oxidizing solutions [7,8,9]. Accordingly, it is often fabricated into membranes, and has been applied in desalination and water treatment [10], oil/water separation [11], as a chromatographic support material [12], and in medical applications [13]. For example, Anari et al. [14] modified ECTFE membrane by use of a simple surface oxidation to induce the production of hydrophilic functional groups (–COOH and –OH) on the membrane surface. The presence of these groups could cause polar/electrostatic interaction. As a result, a lot of salt might deposit on membrane surface to realize seawater desalination. Pan et al. [15] fabricated ECTFE-SiO_2_ hybrid porous membrane with hierarchical micro/nano-structural suface via thermally induced phase separation (TIPs). The results showed that the membrane had high oil/water separation efficiency, and can also be reused many times in various PHs. Alicea et al. [16] attempted to use ECTFEs as support materials for capillary electrochromatography (CEC). In CEC, electroosmotic flow (EOF) colud be produced in capillary columns packed with ECTFE by using acetonitrile–water mobile phases. Furthermore, the potential of ECTFE for CEC was verified by separating a mixture of amino acids. Bosh et al. [13] coated titanium substrate with ECTFE using an electrophoretic deposition process followed by heat treatment to produce the materials suitable for medical applications. In practical applications, the performances of ECTFE depends on its supramolecular structure, which is formed during the processing stage. Thus, this study on the IT and NIT crystallization kinetics of ECTFE can provide powerful theoretical support for its processing.

However, to the best of our knowledge, the crystallization kinetics and morphology of ECTFE has been rarely described in the literature. In this work, the main objective is to fill this gap by investigating the IT (Avrami equation) and NIT crystallization kinetics (Jeziorny, Ozawa, and Mo’s models), crystalline morphology, and the structure of ECTFE in detail.

## 2. Experimental

### 2.1. Raw Materials

**Figure 1:**
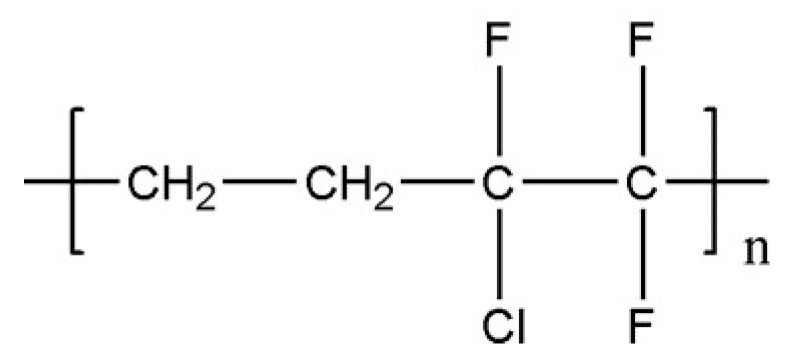
The molecular formula of ECTFE.

Commercial ECTFE resin HALAR^®^ 1400LC (d = 1.68 g·cm^−3^) was purchased from Solvay Specialty Polymers LLC, Chicago, IL, USA. This resin has a melt flow index of 500 g/10 min (230 °C, 21.6 N). To remove any excess moisture, the resin was oven-dried at 80 °C for 12 h. The repeating unit of this polymer is given in Figure 1.

### 2.2. Experimental Details

Isothermal (IT) and non-isothermal (NIT) crystallization kinetics were studied using DSC (DSC 8000, Perkin Elmer, Norwalk, CT, USA). Indium was used as a calibration standard for temperature. In each DSC run, a fresh amount of polymer was used, and the mass of the probe was between 4.5 and 5.5 mg to eliminate the influence of sample size. The probes were loaded into DSC at 25 °C, and liquid N_2_ was used for cooling.

The temperature range for IT crystallization was between 204 °C and 212 °C, with the step size of 2 °C giving five points. Each sample was wrapped inside Al pans, heated at the rate of 10 °C/min to 265 °C, and then maintained at this temperature for 5 min to eliminate all previous thermal effects. This was followed by fast isothermal cooling (200 °C/min) to the target crystallization temperature.

For NIT crystallization tests, the samples were pre-heated to 265 °C with a heating rate of 25 °C/min, maintained for 5 min, and then cooled to target temperatures using the following four cooling rates: 10, 20, 30, and 40 °C/min. As dynamic DSC measurements were limited by the slow response of the instrument furnace and low heat transfer to the samples, the acquired data were carefully evaluated, and the experiments were repeated at least three times.

The XRD patterns were determined using a D8 Discovery X-ray diffractometer (D8 DISCOVERY, Bruker AXS, Karlsruhe, Genman), with a Cu Kα (λ = 1.5406 Å) radiation source in the diffraction angle (2θ) range of 5~50°, and steps of 5°·min^−1^ at 40 kV and 40 mA.

The crystal morphologies of the ECTFE were observed with the WMP-6880 hot stage polarizing microscope (WMP-6880, Wumo Optical Instrument Co., Ltd., Shanghai, China). About 1 mg of the sample was heated from 25 to 255 °C at a heating rate of 50 °C·min^−1^, and held at 255 °C for 5 min to remove the heat history. After that, the sample was cooled at a cooling rate of 10 °C·min^−1^ until 210 °C, and the crystal morphologies of the sample at different temperatures during the cooling process were observed.

## 3. Results and Discussion

### 3.1. Isothermal Crystallization Properties of ECTFE

**Figure 2:**
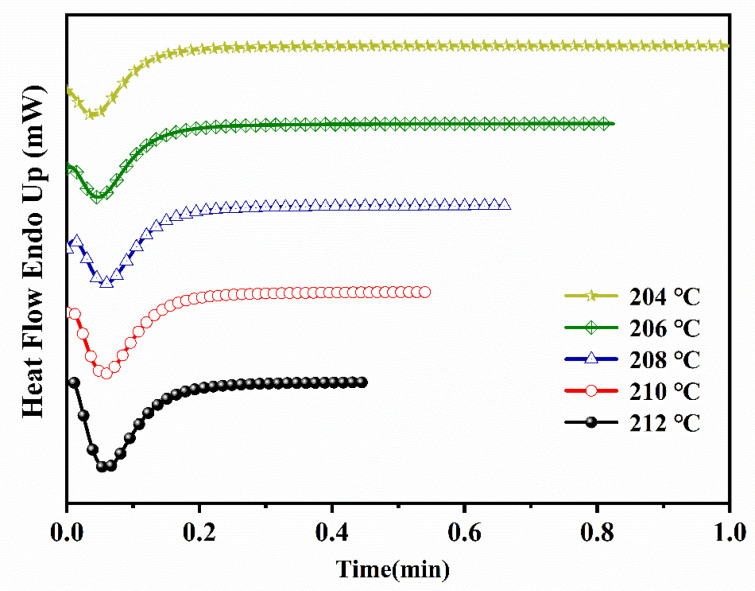
The DSC cooling curves of ECTFE at five target *T_c_*.

The DSC profiles of ECTFE under isothermal crystallization conditions at several crystallization temperatures (*T_c_*) are shown in Figure 2.

**Figure 3:**
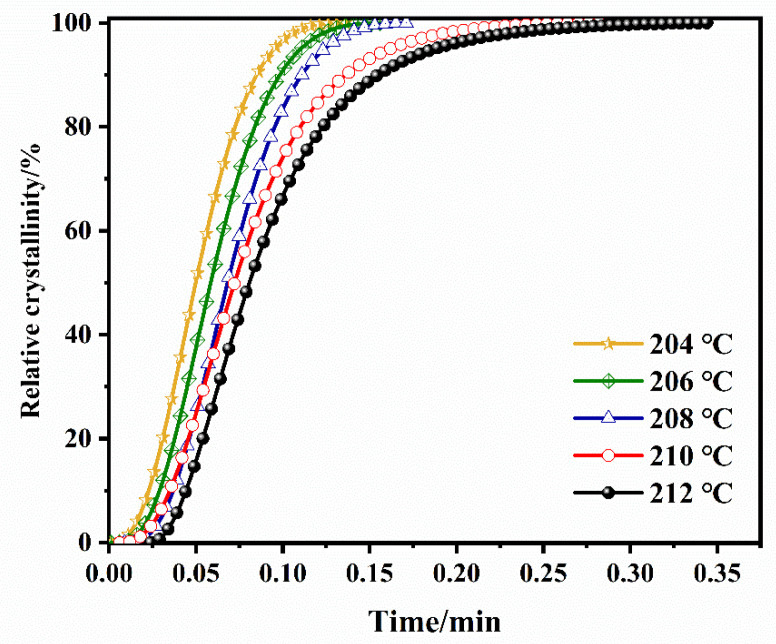
The relative crystallinities of ECTFE polymer derived from DSC cooling curves for five target temperatures.

The peak associated with crystallization was present in all samples, but its width and position were highly dependent on the temperature. At higher *T_c_*, the peak broadened and shifted to longer crystallization times, which confirms that the crystallization rate of ECTFE is decreasing. The degree of crystallinity (*X_t_*) can be calculated from DSC cooling isotherms (Figure 2) under the assumption that *X_t_* is proportional to the heat released during the crystallization using the following formula:

(1) $$X_{t}=\frac{\int_{t_{0}}^{t}\frac{dH}{dt}dt}{\int_{t_{0}}^{\infty }\frac{dH}{dt}dt}$$

where *t*_0_ is the onset crystallization time, *t* is the time of measurement, and *dH*/*dt* is the heat flow rate. The higher relative crystallinity of ECTFE at lower *T_c_* is shown in Figure 3.

#### 3.1.1. The Isothermal Crystallization Kinetics Analyzed Using the Avrami Equation

The general equation that describes the relative degree of crystallinity *X_t_* under the isothermal (IT) crystallization process is as follows:

(2) $$X_{t}=1-exp\left(-\delta_{c}\right)$$

where $\delta_{c}$ is a parameter described by many authors [17,18,19,20,21,22]. Among them, the Avrami equation is the most suitable and most widely used one in polymer crystallization studies. In this equation, $\delta_{c}=Z_{t}t^{n}$, where *n* is the Avrami exponent which reflects the dimensionality of the crystallization process. For uniform nucleation and crystal growth, *n* is equal to D + 1, where D is the dimensionality of crystallization space. Moreover, parameter *Z_t_* is the crystallization rate constant which depends on the type of nucleation and the crystal growth rate. The logarithmic transformation of the Avrami equation gives the following:

(3) $$log\left[-ln\left(1-X_{t}\right)\right]=logZ_{t}+n\log t$$

**Figure 4:**
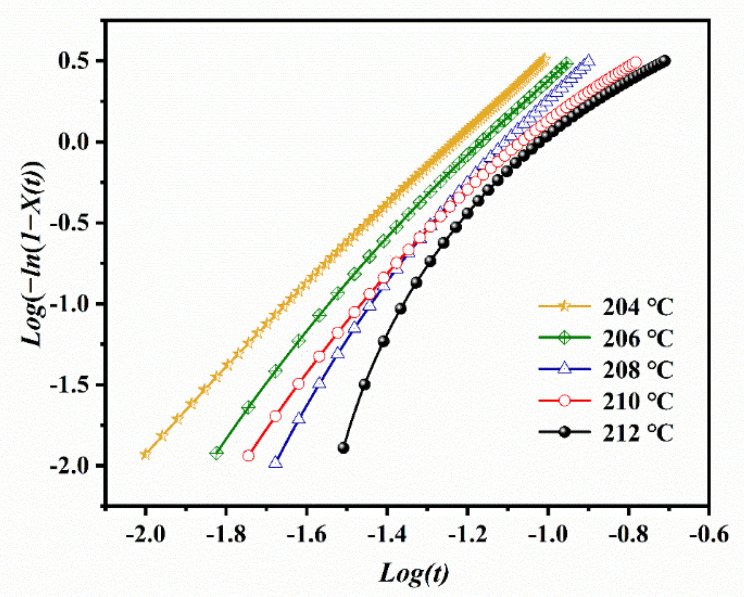
The Avrami plots for the isothermal crystallization of ECTFE at five target crystallization temperatures.

The parameter *n* can be calculated from the slope of $log\left[-ln\left(1-X_{t}\right)\right]$ versus logt plot, while *Z_t_* is the intercept of this plotline. The corresponding plots for ECTFE are given in Figure 4.

The initial stage at each crystallization temperature is linear, and the Avrami equation is applicable for this part of the graph. At longer crystallization times, the curves deviate from the linearity due to sphaerocrystal collision, i.e., the collision of initially formed spherulites in the secondary stages of crystal growth [23,24]. The n and *Z_t_* values calculated from the linear part of the Avrami plots are listed in Table 1. The linearity at each *T_c_* is revealed by Pearson’s correlation coefficients *r*^2^ close to 1, and this confirmed the applicability of Avrami model for the IT crystallization kinetics of ECTFE. The n values were between 2.39 and 3.05, and decreased with the increase in *T_c_*. This corresponds to the homogenous formation of two-dimensional ECTFE at lower *T_c_* and heterogeneous nucleation with three-dimensional spherulite growth at higher *T_c_*. Moreover, the crystallization rate constant *Z_t_* decreased with the increase in *T_c_*, which suggests a shift from homogeneous to heterogeneous nucleation at higher *T_c_*.

The crystallization half-time (*t*_1/2_) is another important parameter of crystallization kinetics and represents the crystallization time at which the relative crystallinity achieves 50% [25]. It is calculated as follows:

(4) $$t_{1/2}={\left(\ln2/Z_{t}\right)}^{1/n}$$

From this equation, the crystallization rate *G*$\;\mathrm{can}\;\mathrm{be}\;\mathrm{expressed}\;\mathrm{as}\;t_{1/2}^{-1}$ [26,27]. Moreover, the IT crystallization of ECTFE was characterized by the time needed for a material to reach its maximum crystallinity (*t*_*max*_). It can be calculated from this assumption that the maximum crystallinity corresponds to the point where the heat flow rate *dH/dt* is equal to 0 [28], as follows:

(5) $$t_{max}={\left(\frac{n-1}{n\times Z_{t}}\right)}^{1/n}$$

The values of *G* and *t_max_* are listed in the Table 1.

**Figure 5:**
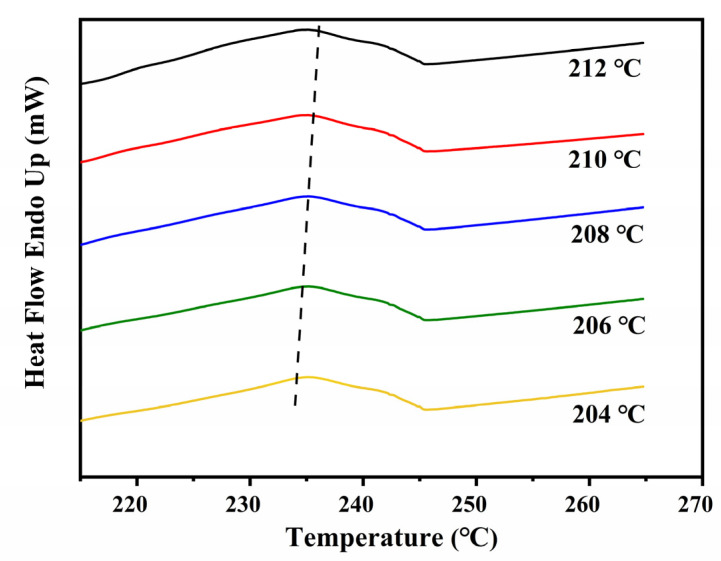
DSC melting behavior of ECTFE crystallized isothermally at five temperatures.

**Figure 6:**
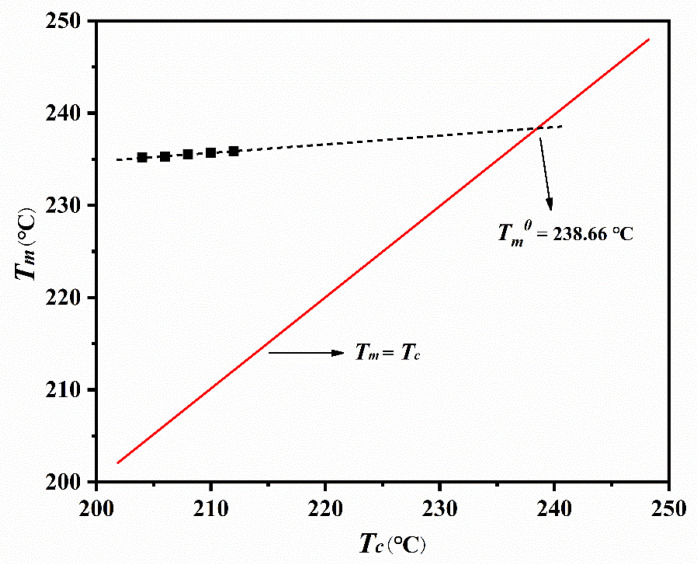
Hoffman–Weeks plot for ECTFE.

The equilibrium melting point ($T_{m}^{0}$) is useful for quantitative and qualitative analysis of the crystallization rate of a flexible, linear polymer. This parameter is related to the melting temperature of an infinitely large crystal, and can be accurately determined from DSC melting curves (Figure 5) using the Hoffman–Weeks theory [29]. According to this theory [29], the $T_{m}^{0}$ can be obtained from the following dependence:

(6) $$T_{m}=\eta T_{c}+\left(1-\eta \right)T_{m}^{0}$$

where $T_{m}$ denotes to the apparent melting point at $T_{c}$, and η is parameter of the stability. The Hoffman–Weeks plot for ECTFE is shown in Figure 6. The plot of *T_m_* vs. *T_c_* provided a straight line, with $T_{m}^{0}$ = 238.66 °C and η = 0.09.

**Figure 7:**
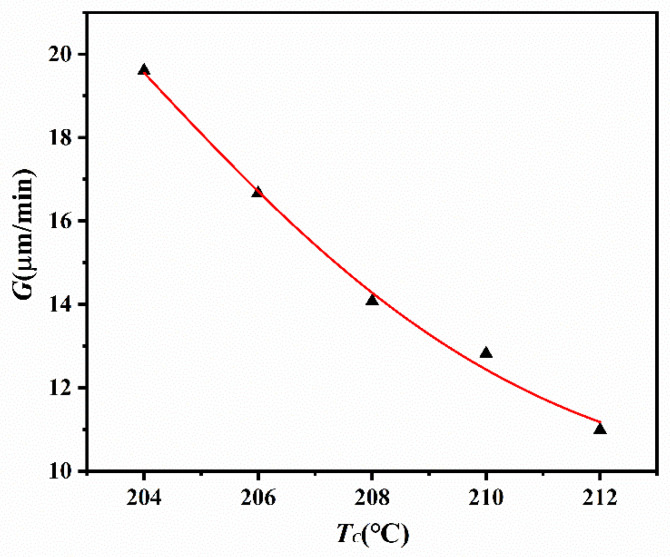
The plot of crystallization rate of ECTFE vs. *T_c_*.

The temperature dependence of crystallization rate *G* is illustrated in Figure 7. An obvious drop in *G* is observed at higher temperatures, and this is a common phenomenon in polymer crystallization.

#### 3.1.2. The Kinetics of Spherulitic Growth for ECTFE

The kinetics of spherulite growth for ECTFE was fitted into the Lauritzen–Hoffman model [30] for secondary nucleation, as follows in Equation (7):

(7) $$G=G_{0}exp\left[-\frac{U^{*}}{R\left(T_{c}-T_{\infty }\right)}\right]exp\left[-\frac{K_{g}}{T_{c}\left(\Delta T\right)f}\right]$$

In this equation, *G*_0_ denotes the pre-exponential factor, U* is the energy barrier for transporting the polymer to the crystallization site, *R* is the universal gas constant, *T*_∞_ represents a temperature that the mobility of polymer chains halts, ΔT is the difference between $T_{m}^{0}$, and $T_{c}$ and describes the degree of supercooling, f is a parameter that corrects the variations in the enthalpy of fusion calculated as $f=2T_{c}/\left(T_{m}^{0}+T_{c}\right),$ and $K_{g}$ is the nucleation constant. The parameter U* can be represented as $U^{*}=\left(C_{1}T_{c}\right)/\left(C_{2}+T_{c}+T_{g}\right)$, where the constants *C*_1_ = 4120 cal mol^−1^ and *C*_2_ = 51.6 K [31]. Hoffman et al. [32] previously stated that U* and *T*_∞_ are universal. The U* values obtained in this work at five temperatures are listed in Table 1, and *T*_∞_ are calculated as $T_{g}-51.6\;K$.

**Figure 8:**
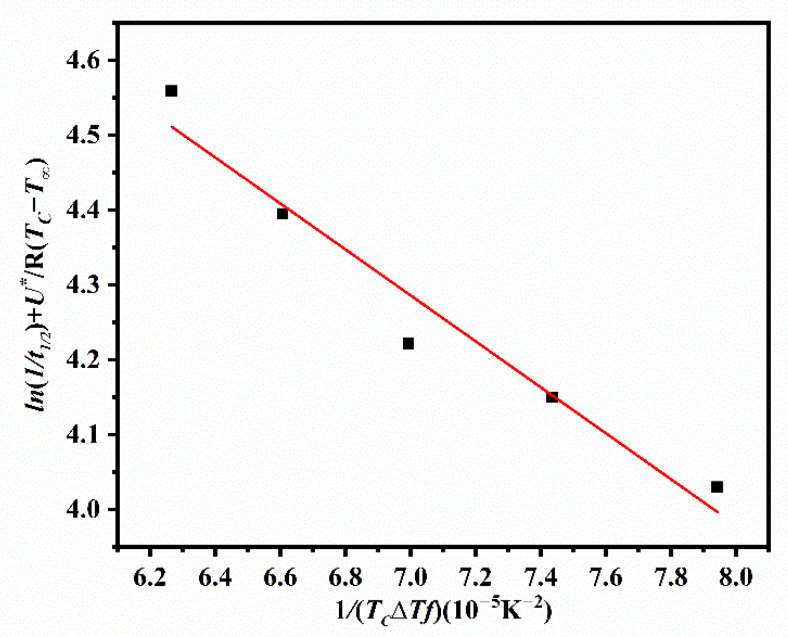
Double-log plot of the Lauritzen–Hoffman model for ECTFE.

The linearization of Equation (7) in the form of double-log model provides Equation (8), as follows:

(8) $$ln\left(\frac{1}{t_{1/2}}\right)=ln{\left(\frac{1}{t_{1/2}}\right)}_{0}-\left(\frac{U^{*}}{R\left(T_{c}-T_{\infty }\right)}\right)-\left(\frac{K_{g}}{T_{c}\left(\Delta T\right)f}\right)$$

where the plot of $ln1/t_{1/2}+U^{*}/R\left(T_{c}-T_{\infty }\right)$ versus $1/T_{c}\Delta Tf$ yields a trendline with the slope $K_{g}$ and intercept $ln{\left(1/t_{1/2}\right)}_{0}$. The statistical quality of the linear fit represents a measure of validity of the Lauritzen–Hoffman model for a given crystallization process. As in Figure 8, this model fitted well with the data for the isothermal (IT) crystallization of ECTFE. The ${\left(1/t_{1/2}\right)}_{0}$. value is 624.476 min^−1^, the $K_{g}$. value is 3.068 × 10^4^ K^2^, and it has a Pearson’s correlation coefficient of 0.939. Therefore, the Lauritzen–Hoffman model is applicable for the quantitative analysis of the spherulitic growth rate of ECTFE.

Activation energy for IT crystallization of ECTFE (Δ*E*) can be obtained from the temperature dependence of the crystallization rate constant (*Z_t_*) using the following form of the Arrhenius Equation (9) [33]:

(9) $$Z_{t}^{1/n}={\left(Z_{t}\right)}_{0}\times exp\left(\frac{-\Delta E}{R\times T_{c}}\right)$$

A simple logarithmic transformation of Equation (9) results in Equation (10), as follows:

(10) $$\frac{1}{n}\times lnZ_{t}=ln{\left(Z_{t}\right)}_{0}-\frac{\Delta E}{R\times T_{c}}$$

**Figure 9:**
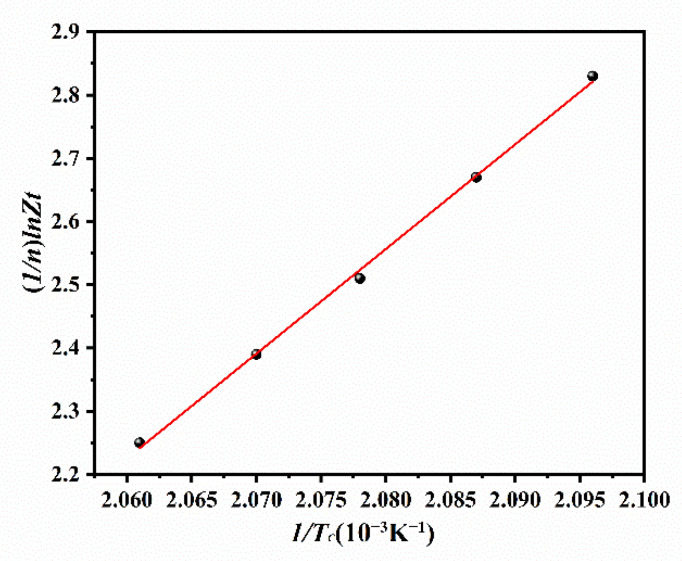
The Arrhenius plot of IT crystallization of ECTFE.

In Equation (10), the slope of the $\frac{1}{n}\times lnZ_{t}$ versus $1/T_{c}$ plot yields Δ*E*, and the intercept is a temperature-independent pre-exponential factor ${\left(Z_{t}\right)}_{0}$. In Figure 9, the calculated Δ*E* was −137.68 kJ/mol. The high correlation coefficient (*r*^2^ = 0.99) confirms the applicability of the Arrhenius model for the determination of Δ*E* for the IT crystallization of ECTFE.

### 3.2. Non-Isothermal Crystallization Properties of ECTFE

**Figure 10:**
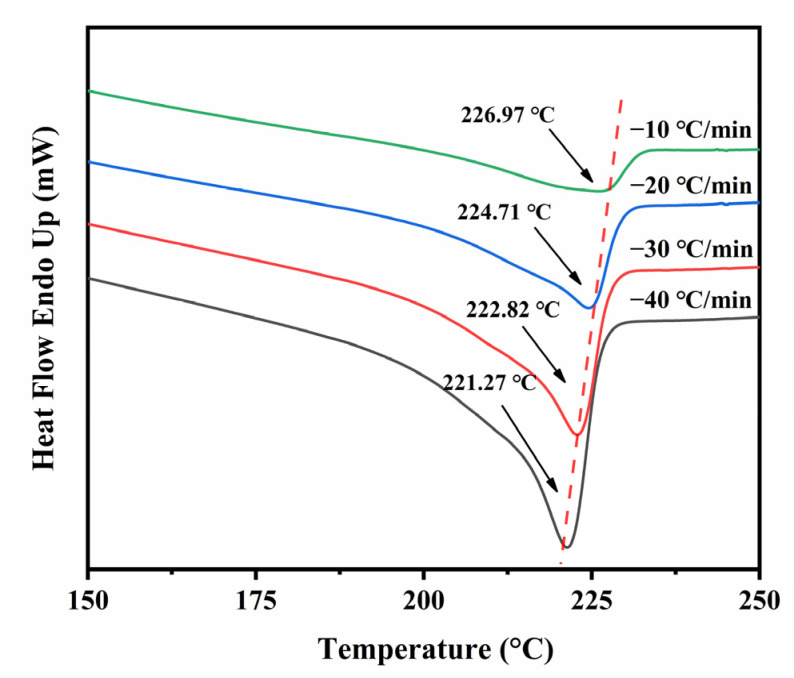
The DSC curves of ECTFE at four cooling rates.

We also studied the crystallization of ECTFE under non-isothermal (NIT) conditions, as these data are important for the optimization of melt extrusion processes. The crystallization of polymers at various cooling rates is characterized by the initial crystallization temperature (*T_i_*), peak temperature (*T_p_*), end temperature (*T_e_*), the melting enthalpy (Δ*H_c_*), and the degree of crystallinity (*X_c_*). The data for ECTFE are obtained from DSC curves at four cooling rates between 10 and 40 °C·min^−1^ (Figure 10), and these are listed in Table 2.

The *X_c_* value was calculated from the Equation (11), as follows:

(11) $$X_{c}=\frac{\Delta H_{c}}{\Delta H_{c}^{0}}\times 100\%$$

where Δ*H_c_* is the experimental melting enthalpy, and Δ$H_{c}^{0}=$ 40 J/g [34] is the standard melting enthalpy of ECTFE.

The trends $T_{p}$ and Δ*H_c_* closely describe the influence of cooling rate on the crystallization capacity of the polymer. According to the DSC curves shown in Figure 10, the increase in cooling rate decreases the *T_p_* (Table 2). This trend may be explained by the increased number of ECTFE chains that are frozen in their original positions at higher cooling temperatures, which results in nucleation hysteresis. Accordingly, *T_i_* increases at lower cooling rates. In addition, Δ*H_c_* and *X_c_* of ECTFE decrease with the increase in cooling rate.

#### 3.2.1. Polarized-Light Microscopy (POM) Reveals the Morphology of ECTFE Spherulites Formed during Non-Isothermal Crystallization

**Figure 11:**
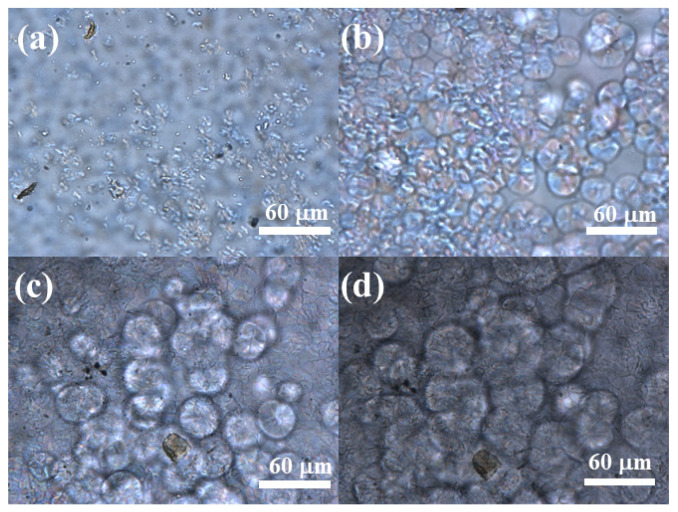
The POM images of ECTFE (**a**) at initial crystallization temperature, (**b**) between the initial and crystallization peak temperature, (**c**) at crystallization peak temperature, and (**d**) at the end crystallization temperature.

Spherulites are spherical, highly ordered, lamellar structures with higher density and hardness compared with the amorphous regions of a polymer. The details about the morphology and growth rate of spherulites are important for the control of the physical properties of a final product. Figure 11 shows the POM images of ECTFE. When the sample is cooled at the rate of 10 °C·min^−1^ until 210 °C, the sample begins to crystallize in the cooling process. The results show the presence of spherulites in the cooling processing. Spherulites are organized in a hexagonal crystal system with a circular cross-section. These structures are formed due to numerous nucleation sites and the rapid cooling of molten polymer that prevents regular crystal growth. To reduce the surface energy, crystals grow radially and form crystals with a circular cross-section [35].

#### 3.2.2. Wide-Angle X-ray Diffraction (XRD) Confirm Hexagonal Crystal System for ECTFE

**Figure 12:**
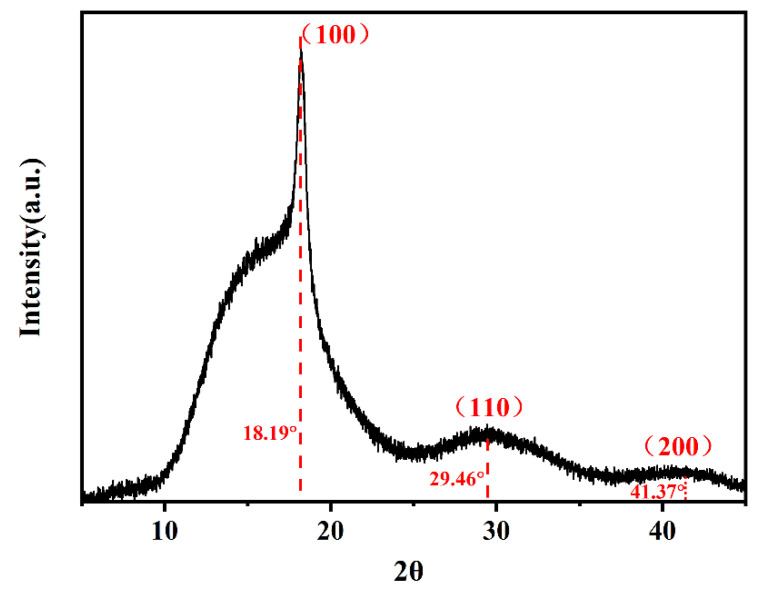
The XRD patterns of ECTFE.

X-ray diffraction was used as an additional tool to analyze the degree of crystallinity *X_T_* and the morphology of the ECTFE polymer formed under NIT conditions. The *X_T_* value of 58.06% is consistent with the DSC data (Table 2). The ECTFE exhibits three distinct XRD diffraction peaks at 18.19°, 29.46°, and 41.37° (Figure 12). The calculated ratio of sin 2θ values is approximately 1:3:4, which corresponds to (100), (110), and (200) planes of hexagonal crystal system, as already reported in the literature [34].

### 3.3. Non-Isothermal Crystallization Kinetics of ECTFE

Technological processing, such as molding and melt spinning, apply different cooling rates to the polymer [36,37,38,39]. Therefore, it is useful to study the non-isothermal (NIT) crystallization kinetics of ECTFE. In this work, the crystallization data were fitted into the following three kinetic models: Avrami equation modified by Jeziorny, Ozawa, and Mo’s models [40].

**Figure 13:**
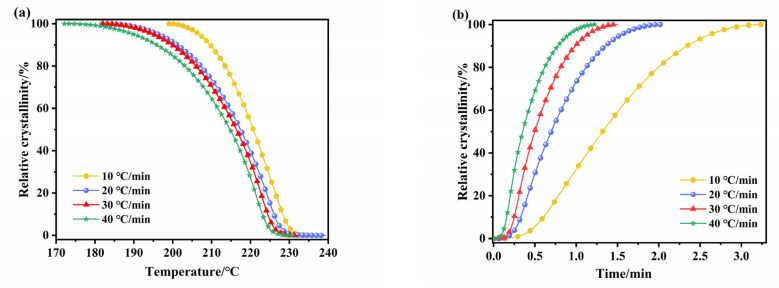
The temperature profile (**a**) and time profile (**b**) of the relative crystallinity of ECTFE.

In the case of NIT crystallization, the relative crystallinity is a function of cooling temperature and can be calculated as follows:

(12) $$X_{T}=\int_{T_{o}}^{T}\left(\frac{dH_{C}}{dT}\right)dT/\int_{T_{o}}^{T_{f}}\left(\frac{dH_{C}}{dT}\right)dT$$

where *T_o_*, *T* and *T_f_* denote onset, arbitrary, and final temperatures of crystallization, respectively. The heat change with the infinitesimal change in temperature is given as *dH_c_*/*dT*. All *X_T_* temperature profiles exhibit the reversed S-shape (Figure 13a). This indicates the fast formation of nuclei from polymer melt in the initial stage of crystallization that slows down during the crystal growth stage. The shift of the *X_T_* temperature profile to the lower temperatures shows that the degree of crystallinity is reducing with the increase in cooling rate. At high cooling rates, the molecules of ECTFE polymer in the liquid phase do not have enough time to rearrange into more regular structures, resulting in a larger number of nucleation sites and crystallization at lower temperatures.

The temperature profile of relative crystallinity of a polymer under NIT conditions can be transformed into the time-dependent profile for each *X_T_* using the following equation:

(13) $$t=\frac{T_{0}-T}{\Phi }$$

where *T*_0_ is the onset crystallization temperature, *T* is the temperature at the time *t*, and *φ* denotes the cooling rate. The time needed for a polymer to reach 50% relative crystallinity is defined as *t*_1/2_. The *t*_1/2_ values for ECTFE at each cooling rate are calculated from *X_T_* time curves (Figure 13b) and are listed in Table 3. The increase in *φ* from 10 to 40 °C/min reduces *t*_1/2_ from 1.320 to 0.351 min. These results confirm faster crystallization of ECTFE at higher cooling rates.

#### 3.3.1. Analysis of Non-Isothermal Crystallization Kinetics of ECTFE Using the Avrami Model Modified by Jeziorny

To account for temperature changes during the crystallization, Jeziorny [41,42] proposed a modification of the Avrami model (Equation (3)) where parameter *Z_t_* is corrected for cooling rate *φ*, giving a modified growth rate constant of *Z*_*c*_ [43], as follows:

(14) $$\log Z_{c}=\frac{\log Z_{t}}{\Phi }$$

**Figure 14:**
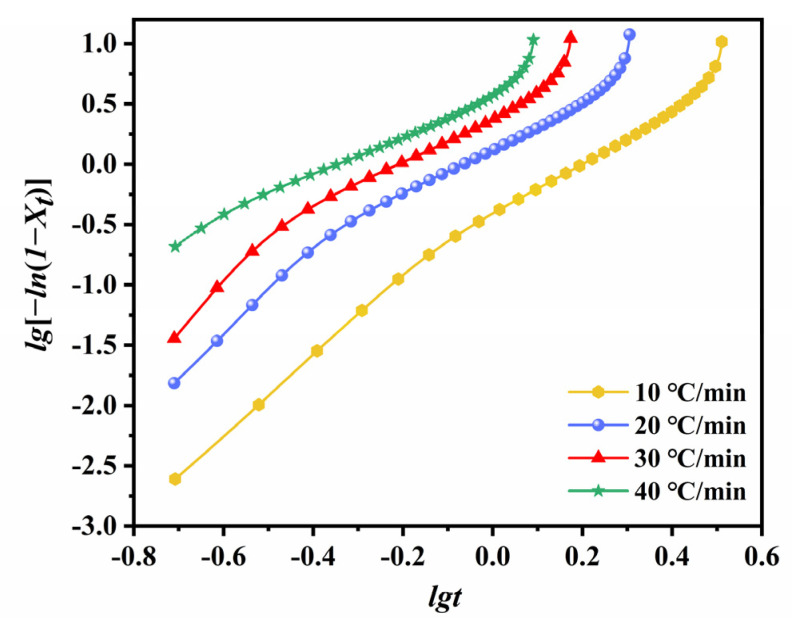
Modified Avrami plots of $log\left[-ln\left(1-X_{t}\right)\right]$ versus logt.

Double-log plots of the modified Avrami equation (Figure 14) yield a straight line in the early stages of crystallization. In the secondary stages, the plot deviates from the linearity due to the collisions of the initially formed spherocrystals.

The statistical parameters obtained by the linear fitting of the early stages of crystallization are listed in Table 3. The Avrami exponent n as a measure of uniform crystal growth decreases from 3.37 for the lowest cooling rate to 1.64 for the highest cooling rate. We conclude from the non-integer values of *n* that the non-isothermal (NIT) crystallization of ECTFE is a mixed-type growth with variable nucleation and significant effects of secondary crystallization. It is noteworthy that the values of *Z_t_* and *n* in the NIT crystallization do not have the same physical meaning as in the IT crystallization due to constant changes in temperature. Nevertheless, they still provide insights into the kinetics of the NIT crystallization of ECTFE [44].

#### 3.3.2. Non-Isothermal Crystallization Kinetics Based on Ozawa Model

The Ozawa model [45] is based on Evans’ theory, and treats NIT crystallization as the sum of infinitesimal isothermal crystallization processes. Considering the nucleation and crystal growth, the Avrami equation can be modified to obtain Equation (15), as follows:

(15) $$1-X_{T}=\exp\left(-K_{T}/\varphi^{m}\right)$$

and its double-log form, as follows:

(16) $$log\left[-ln\left(1-X_{T}\right)\right]=logK_{T}-mlog\varphi$$

**Figure 15:**
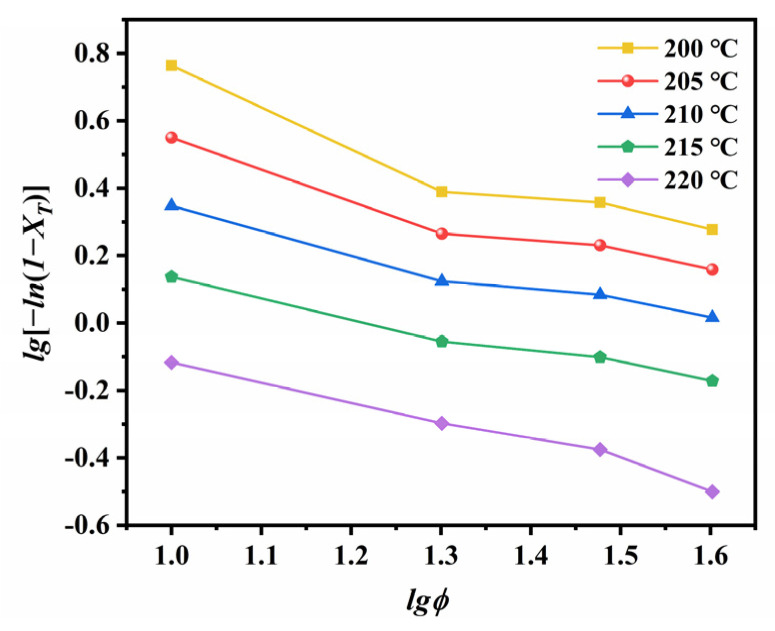
Ozawa plots for the NIT crystallization of ECTFE.

The parameter *K_T_* is the cooling function at a given temperature *T*, and m represents the Ozawa exponent that reflects the type and mechanism of nucleation. By plotting the crystallization data according to Equation (16) in the temperature range from 200 to 220 °C (Figure 15), we obtained the values for *K_T_* and m (Table 4).

**Figure 16:**
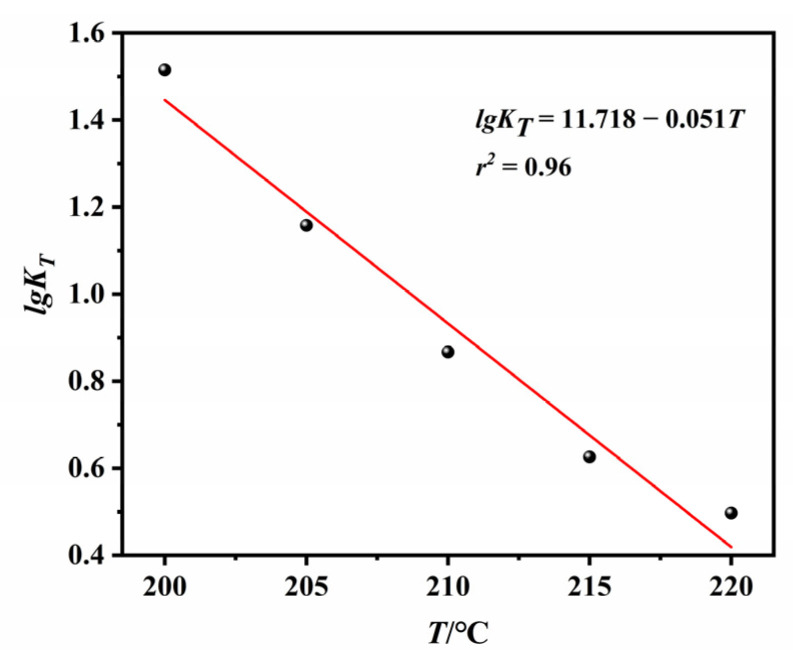
Plot of $logK_{T}$ versus *T* for NIT crystallization of ECTFE.

The negative slope of the Ozawa plot suggests that the relative crystallinity at a given temperature increases with the increase in cooling rate. The variation in m suggests that the crystallization mechanism changes with temperature. According to theory, the dimensionality of crystal growth changes continuously in the early stages of crystallization due to particle collisions. In the later stages, the system reaches an equilibrium, and the m value remains constant until the completion of crystallization [46]. The temperature dependence of cooling factor *K_T_* is shown in Figure 16. The excellent linearity of Figure 15 and Figure 16 confirm that the crystallization of ECTFE under NIT conditions can be described well by the Ozawa model.

#### 3.3.3. Non-Isothermal Crystallization Kinetics Based on Mo Model

Mo [47] developed a model for non-isothermal (NIT) crystallization kinetics that combines the Avrami and Ozawa equations and used it for studying the crystallization kinetics of PA66, PA11, and PA4. This model can be expressed as follows:

(17) logφ=logG(T)−αlogt

where $G\left(T\right)={\left[K_{T}/Z_{t}\right]}^{1/m}$ represents a required cooling rate to attain a certain degree of crystallinity within the unit crystallization time, and α is the ratio of Avrami exponent *n* and Ozawa exponent *m* (α=n/m).

**Figure 17:**
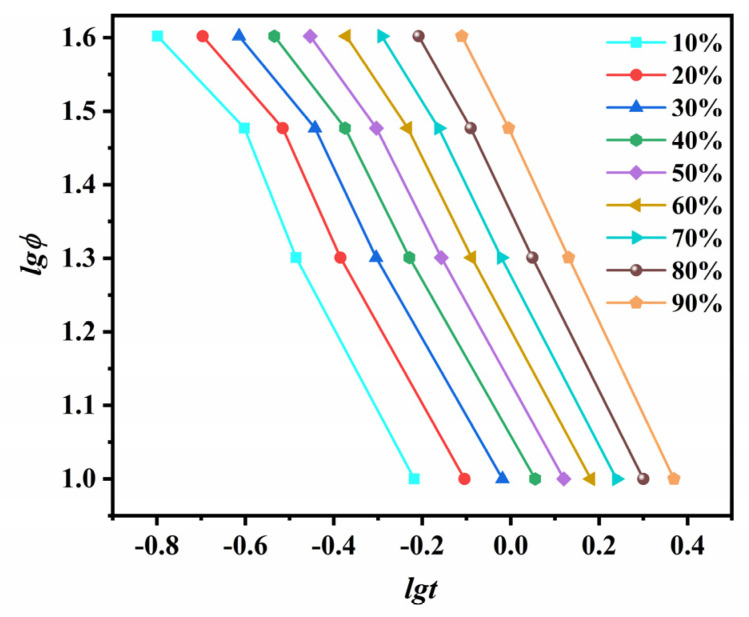
Mo’s plot for the NIT crystallization kinetics of ECTFE at the given *X_T_* values.

Mo’s plot for the NIT crystallization kinetics of ECTFE is shown in Figure 16. The high linearity of logφ versus logt plot, especially at higher degrees of crystallinity, illustrates that Mo’s model is adequate for describing the NIT crystallization kinetics of ECTFE. The *G(T)* and *α* were determined from the intercept and the slope of Figure 17, respectively, and were listed in Table 5. The results show that *G(T)* increases significantly at higher *X_T_* values, and this trend might be explained by certain difficulties in ECTFE crystallization to a high-crystalline state. Moreover, *α* values are constantly between 1.03 and 1.26 for the entire range of *X_T_* values. This result validates the applicability of Mo’s model for describing the NIT crystallization kinetics of ECTFE.

#### 3.3.4. Crystallization Activation Energy (Δ*E*)

Activation energy (Δ*E*) for the non-isothermal crystallization of a polymer can be calculated from the Kissinger equation [48]. This model associates the changes in the cooling rate (*φ*) and crystallization peak temperature (*T_p_*) with Δ*E* according to the following equation:

(18) $$\frac{d\left[ln\left(\Phi /T_{p}^{2}\right)\right]}{d\left(1/T_{p}\right)}=-\frac{\Delta E}{R}$$

**Figure 18:**
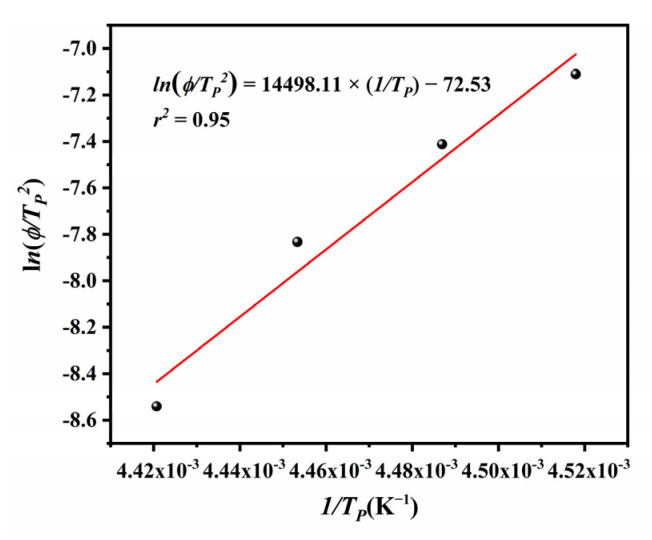
Kissinger plots for the evaluation of crystallization activation energy.

The crystallization activation energy was −120.54 kJ/mol, calculated as the slope of ln(*φ*/*T*_P_^2^) versus 1/*T*_P_ plot (Figure 18). Compared with the Δ*E* value of high-density polyethylene (HDPE), −306.5 kJ/mol [49], the crystal growth of ECTFE is much easier. This can be explained by the facilitated folding of ECTFE molecular chains imposed by the addition of larger and asymmetric trifluorovinyl chloride groups.

## 4. Conclusions

In this paper, the isothermal (IT) and non-isothermal (NIT) crystallization kinetics and morphology of ECTFE were investigated, as these parameters are important for the processability of a polymer. The DSC provided the equilibrium melting point, degree of crystallinity, and the activation energy of crystallization. The data for IT crystallization fitted well with the Avrami model. Additionally, Avrami exponent *n* values were between 2.39 and 3.05, and decreased with the increase in *T_c_*, which corresponds to the homogenous formation of two-dimensional ECTFE at lower *T_c_* and heterogeneous nucleation with three-dimensional spherulite growth at higher *T_c_*. The equilibrium melting point for ECTFE was found to be 238.66 °C. The activation energy for IT crystallization was determined as −137.68 kJ/mol. Polarized optical microscopy and wide-angle XRD revealed the presence of ECTFE spherulites organized in a hexagonal crystal system. These results also indicated that secondary crystallization caused by the collision of the ECTFE spherulites influenced the morphology, crystal growth, and crystallization kinetics. The Jeziorny model could only be used to describe the primary stage of NIT crystallization. The relative crystalline degree of ECTFE under NIT conditions was about 54.55%, and this decreased with the increase in cooling rate. In addition, Ozawa and Mo’s models were suitable for describing NIT crystallization of ECTFE. Moreover, the higher cooling rates increased the crystallization rate and decreased the crystallization temperature of ECTFE. The activation energy for NIT crystallization was determined as −120.54 kJ/mol. This study will be useful for future optimizations of the extrusion and molding processes of ECTFE polymer.

## Figures and Tables

**Table 1:** The parameters of IT crystallization of ECTFE obtained from the Avrami model.

*T_c_* (°C)	*N*	*logZ*_t_	*r* ^2^	*t*_1/2_ (min)	*t*_*max*_ (min)	*G* (min^−1^)	U* (J/mol)
204	2.40	2.95	0.99	0.051	0.047	19.61	2216.53
206	2.63	3.05	0.99	0.060	0.058	16.67	2220.81
208	2.97	3.25	0.99	0.071	0.070	14.08	2225.08
210	2.34	2.43	0.98	0.078	0.072	12.82	2229.32
212	2.45	2.39	0.95	0.091	0.085	10.99	2233.55

**Table 2:** The DSC data of the ECTFE at four cooling rates.

Cooling Rate (°C/min)	*T_i_* (°C)	*T_p_* (°C)	*T_e_* (°C)	Δ*H_c_* (J/g)	*X_c_* (%)
10	234.01	226.97	202.87	21.82	54.55
20	231.31	224.71	204.76	20.95	52.37
30	230.05	222.82	206.75	19.32	48.30
40	229.86	221.27	206.93	18.89	47.23

**Table 3:** The modified Avrami parameters for the NIT crystallization of ECTFE.

*φ*/(°C/min)	*t*_1/2_ (min)	*N*	*logZ_t_*	*Z_c_*	*r* ^2^
10	1.320	3.37	−0.23	0.95	0.988
20	0.697	3.10	0.48	1.06	0.981
30	0.497	2.63	0.64	1.05	0.979
40	0.351	1.64	0.57	1.03	0.981

**Table 4:** Ozawa parameters for the NIT crystallization of ECTFE.

*T*/℃	*m*	$logK_{T}\;$	*r* ^2^
200	0.79	1.51	0.874
205	0.64	1.16	0.905
210	0.54	0.87	0.940
215	0.50	0.63	0.966
220	0.61	0.50	0.976

**Table 5:** Mo’s parameters for the NIT crystallization kinetics of ECTFE.

$X_{T}/\%$	*α*	G(T)	*r* ^2^
10	1.06	6.09	0.97
20	1.04	7.97	0.98
30	1.03	9.74	0.99
40	1.04	11.58	0.99
50	1.07	13.60	0.99
60	1.10	15.99	0.99
70	1.15	18.93	0.99
80	1.19	22.90	0.99
90	1.26	29.26	0.99

## Data Availability

Not applicable.
